# Fungi of the Murine Gut: Episodic Variation and Proliferation during Antibiotic Treatment

**DOI:** 10.1371/journal.pone.0071806

**Published:** 2013-08-19

**Authors:** Serena Dollive, Ying-Yu Chen, Stephanie Grunberg, Kyle Bittinger, Christian Hoffmann, Lee Vandivier, Christopher Cuff, James D. Lewis, Gary D. Wu, Frederic D. Bushman

**Affiliations:** University of Pennsylvania School of Medicine, Department of Microbiology, Philadelphia, Pennsylvania, United States of America; Leibniz Institute for Natural Products Research and Infection Biology- Hans Knoell Institute, Germany

## Abstract

Antibiotic use in humans has been associated with outgrowth of fungi. Here we used a murine model to investigate the gut microbiome over 76 days of treatment with vancomycin, ampicillin, neomycin, and metronidazole and subsequent recovery. Mouse stool was studied as a surrogate for the microbiota of the lower gastrointestinal tract. The abundance of fungi and bacteria was measured using quantitative PCR, and the proportional composition of the communities quantified using 454/Roche pyrosequencing of rRNA gene tags. Prior to treatment, bacteria outnumbered fungi by >3 orders of magnitude. Upon antibiotic treatment, bacteria dropped in abundance >3 orders of magnitude, so that the predominant 16S sequences detected became transients derived from food. Upon cessation of treatment, bacterial communities mostly returned to their previous numbers and types after 8 weeks, though communities remained detectably different from untreated controls. Fungal communities varied substantially over time, even in the untreated controls. Separate cages within the same treatment group showed radical differences, but mice within a cage generally behaved similarly. Fungi increased ∼40-fold in abundance upon antibiotic treatment but declined back to their original abundance after cessation of treatment. At the last time point, *Candida* remained more abundant than prior to treatment. These data show that 1) gut fungal populations change radically during normal mouse husbandry, 2) fungi grow out in the gut upon suppression of bacterial communities with antibiotics, and 3) perturbations due to antibiotics persist long term in both the fungal and bacterial microbiota.

## Introduction

The effects of antibiotic use on the human microbiome can be challenging to study–confounding factors include complications of the underlying diseases states and concomitant use of additional forms of therapy [1]. Despite these difficulties, outgrowth of fungi has been repeatedly linked to antibiotic treatment at multiple body sites [2]–[8]. Fungal infection associated with antibiotic use is of particular concern in immunocompromised states such as HIV/AIDS [9]–[11], some cancers [8], [12], [13], and transplantation [14]–[18]. Many of these conditions necessitate the use of corticosteroids, which further predisposes the host to fungal infection [19]. Invasive fungal infections have been increasing in recent decades [1], [12], and the rise of azole-resistant species of *Candida* [7], [20], *Aspergillus* [21], [22], and *Cryptococcus* [9], [23] brings further urgency to understanding the interaction between commensal fungi and bacteria during antibiotic treatment.

Rodent models have been used to study the effects of antibiotics on the mammalian gut, using culture based [24], [25], metagenomic [26], and immunologic [26], [27] methods. Antibiotic treatment can predispose the host to infection by pathogens [28], [29] and alter microbial communities long term [29]. Induced exposure to *Candida albicans* shapes the bacterial composition of the murine gut during antibiotic recovery [25] and can cause gastritis [30], while *Candida tropicalis* has been associated with increased severity in ulcerative colitis [31]. Phenotypic effects have been found even after treatment with subclinical doses of antibiotics [32]. In studies of the role of the vertebrate microbiome in mice, antibiotic treatment is often used to suppress the host bacteria, but the effect of this intervention on fungi is not commonly considered [28], [33]–[35].

Here we characterize the bacterial and fungal microbiota of mice during antibiotic treatment and subsequent recovery after cessation, analyzing both the amounts and types of microbes present. We found that fungi indeed grew out upon antibiotic treatment. After cessation of antibiotic treatment, fungal and bacterial communities approached their pre-antibiotic states, but increased abundance of *Candida* persisted in the gut at the last time point studied eight weeks later. To our surprise, we also found that the fungal communities changed radically over time in both control and treated mice. For each condition, specific fungi colonized multiple mice in the same cage, then gave way to different fungal colonists over time, and different patterns were seen in different cages.

## Results

### Longitudinal Analysis of the Murine Gut during Antibiotic Treatment

**Figure 1:**
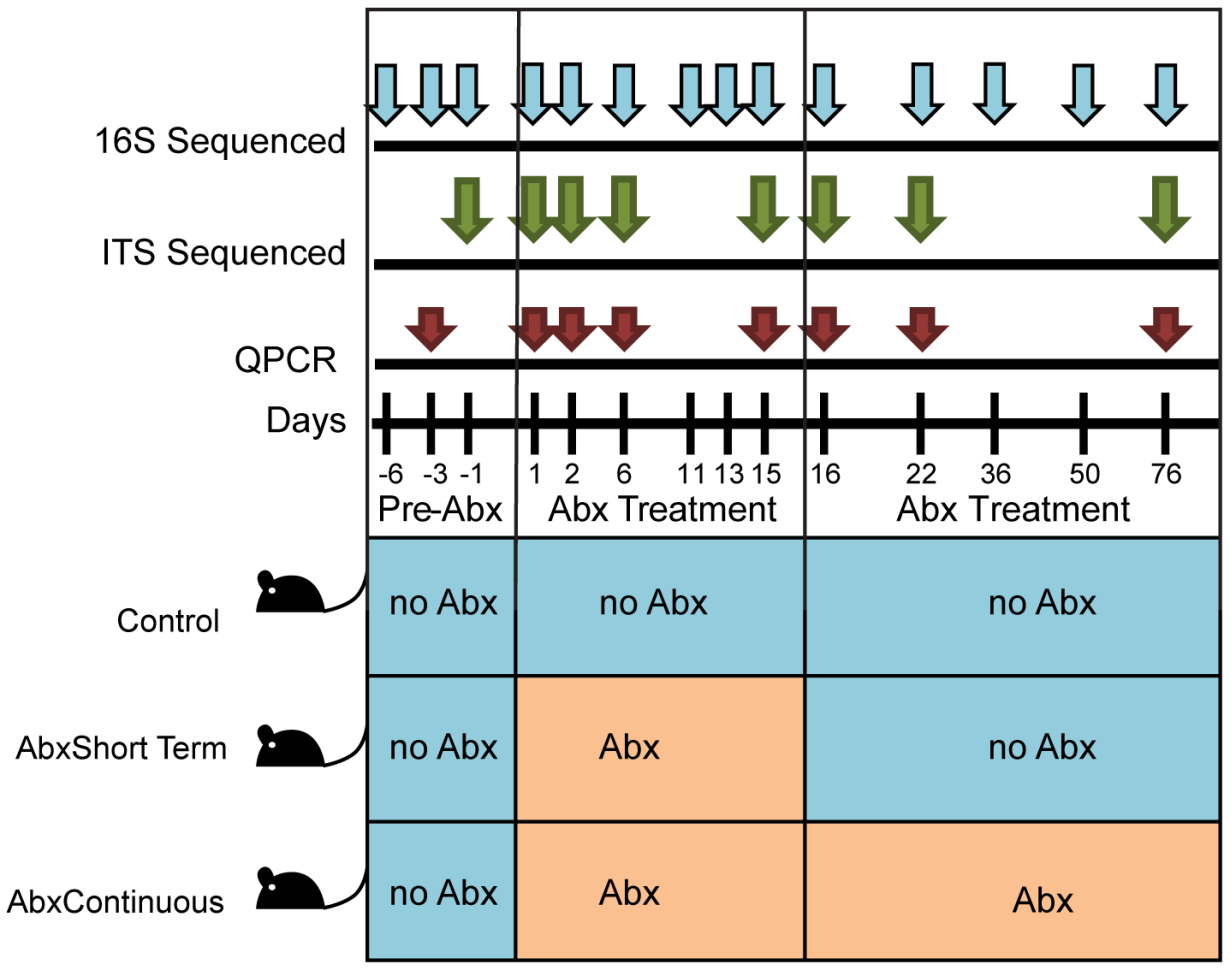
Diagram of the experiment. The time line for the 76 days of sample collection is shown along the top, and the periods of antibiotic treatment are shown at the bottom. Antibiotic treatment was initiated at time zero.

An antibiotic cocktail containing vancomycin, ampicillin, neomycin, and metronidazole was given to twenty C57B6 mice in water. After 2 weeks, antibiotic treatment was stopped for ten of the mice. These mice did not receive any antibiotics during the remaining nine weeks of the study (“AbxShortTerm” mice). The remaining ten mice under antibiotic treatment continued to receive antibiotics for the duration of the study (“AbxContinuous” mice). Ten control mice received no antibiotics over the course of the study (“Control” mice). Fecal samples were collected over one week prior to initiating the study, then at the indicated time points during the study (Figure 1). DNA was purified from stool pellets using a procedure that included bead beating and a high temperature incubation to facilitate lysis of fungal cells [36].

### Analysis of the Numbers of Bacterial 16S and Eukaryotic 18S Gene Copies Present after Antibiotic Treatment

We first investigated the changes in abundance of bacteria and fungi, using stool specimens as a proxy for the lower intestinal microbiome. To assess changes in abundance, we first quantified the relative abundance of bacterial and fungal genomes in the samples per ng of DNA using quantitative PCR. For bacteria, a QPCR assay was used that detected the bacterial 16S rRNA gene, and for fungi, an assay was used detecting the 18S rRNA gene. The primers for the fungal assay were designed to suppress amplification of metazoan DNA originating from the host or food materials [37]. The specificity was confirmed by pyrosequencing products of amplification with these primers (below and Figure S1).

**Figure 2:**
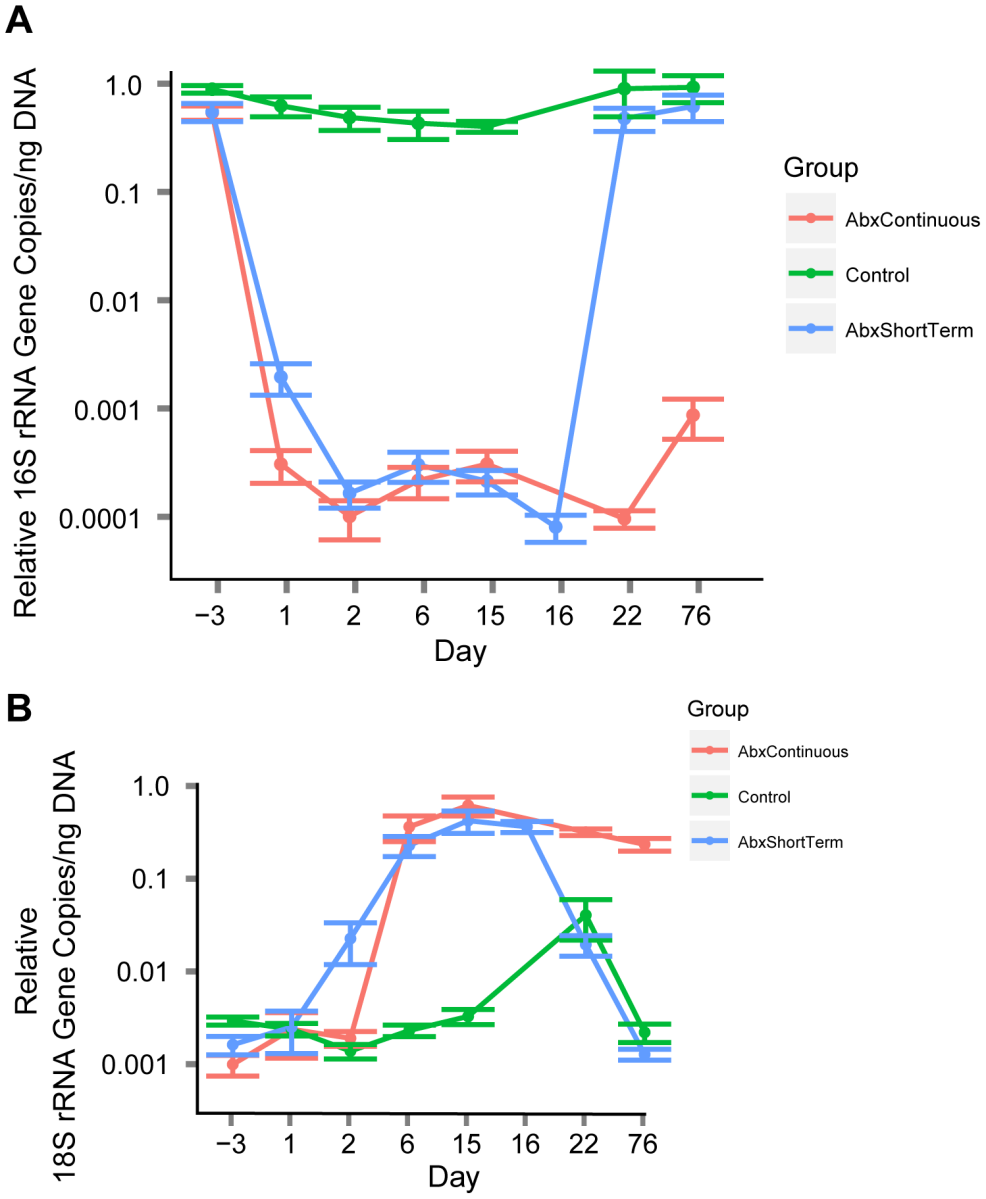
Relative microbial abundance inferred from QPCR. A) Longitudinal analysis of 16S rRNA gene copies per ng of stool DNA. The groups of mice tested are shown by the color code (key at right). Error bars indicate standard error. B) Longitudinal analysis of 18S rRNA gene copies per ng of stool DNA. The groups of mice tested are shown by the color code (key at right). Error bars indicate standard error. The amplicon used was designed to suppress amplification of DNA from mouse or food materials.

At the start of the study, fecal pellets contained high levels of bacterial 16S rRNA genes per ng DNA (∼10^6^ apparent copies; Figure 2A). After initiation of antibiotic treatment, this fell as low as ∼10^2^ apparent copies per ng DNA. Upon cessation of treatment the community recovered to its former numbers. For fungi, prior to antibiotic treatment, ∼10^5^ apparent 18S rRNA gene copies were detected per ng DNA (Figure 2B). Upon initiation of antibiotic treatment the number climbed to between 3–6×10^8^ apparent copies per ng DNA. Upon cessation of antibiotic treatment, the numbers dropped back to roughly their former levels The abundance of fungi in the Control group showed an unexpected increase at day 22. Further analysis showed that the increase was in only one of the two cages housing the control animals, and correlated with the appearance of a new fungal lineage at high levels in all animals in that cage (described below).

Additional information is required to relate these numbers of rRNA gene copies to the numbers of organisms present. This issue is addressed in the next section.

### Assessing the Absolute Abundance of Bacteria and Fungi

Several corrections are required to link the QPCR data to the total number of organisms per stool pellet. One consideration is that bacterial [38], [39] and fungal [40], [41] genomes typically contain multiple rRNA gene copies. From published data on complete genome sequences, we estimated the mean number of 16S rRNA gene copies per bacteria at 5 [38], and 18S copies per fungal genome at 100 [41], though the number for fungi is tentative due to the difficulty of accurately sequencing tandem direct repeats and variability in copy number.

Another concern in assessing possible fungal outgrowth during antibiotic treatment is that the total number of microbes in pellets, and thus total DNA, may go down with treatment, so that fungi could falsely appear to proliferate only because total DNA content went down as bacterial numbers fell. Thus we sought to correct the above assays, which were normalized to weight of DNA, to better reflect the counts of individual organisms by putting the final analysis on a per pellet basis. Average wet weights of pellets were 16.08 mg (SD = 3.329) in the presence of antibiotic (n = 20) and 18.64 (SD = 2.685) in the control mice (n = 19), a slight but significant difference (p = 0.0129, Mann-Whitney U test). A comparison of dry weights showed no significant difference between antibiotic treated (n = 5, mean = 10.615 mg, SD = 1.635791) and control mice (n = 5, mean = 10.875 mg, SD = 0.781025). Thus in what follows we treated the starting weights as equal.

DNA yields per pellet differed substantially (Table 1). Quantification of yields after 15 or 76 days of antibiotic treatment showed drops of 4.7 and 5.7 fold (p = 5.7×10^−5^, Mann-Whitney U test). After withdrawal of antibiotic treatment (AbxShortTerm, Day 76), the total DNA yield returned to within a factor of two of the starting value. Evidently bacterial DNA is the predominant source of DNA in mouse pellets, and the community mostly returned to its former size after cessation of antibiotic treatment. Thus the analysis of the numbers of microbial genomes needs to take into account the drop in total DNA. In addition, we also corrected for inefficiencies in the Taqman detection of 16S rRNA gene copies, which arise because some 16S rRNA gene sequences contain mismatches within the probe binding sites (described in the Methods).

**Table 1:** DNA yields and numbers of genomes inferred from data on ribosomal gene copies.

Sample	DNA yieldper pellet;average (ng)	DNA yieldper pellet(ng); (SD)	Number ofBacterialgenomes/pellet;average	Number ofBacterial genomes/pellet;(SD)	Number of micro-eukaryotegenomes/pellet; average	Number of micro-eukaryotegenomes/pellet; SD	MicroeukaryoteProportion
Control (Baseline)	1.08E+03	4.47E+02	2.17E+09	1.18E+09	2.95E+06	1.25E+06	0.001
Control (Day 15)	6.27E+02	4.02E+02	6.03E+08	5.48E+08	1.98E+06	1.30E+06	0.003
Control (Day 76)	4.50E+02	2.72E+02	6.51E+08	4.97E+08	1.12E+06	9.43E+05	0.002
AbxShortTerm (Baseline)	4.51E+02	1.56E+02	5.79E+08	3.43E+08	7.11E+05	4.43E+05	0.001
AbxShortTerm(Day 15)	1.13E+02	7.42E+01	5.43E+04	4.92E+04	3.56E+07	1.38E+07	0.998
AbxShortTerm(Day 76)	3.94E+02	1.88E+02	3.85E+08	3.12E+08	5.35E+05	4.16E+05	0.001
AbxContinuous (Baseline)	4.39E+02	1.79E+02	5.55E+08	3.68E+08	4.62E+05	1.96E+05	0.001
AbxContinuous(Day 15)	1.48E+02	7.32E+01	9.43E+04	6.53E+04	7.86E+07	3.85E+07	0.999
AbxContinuous(Day 76)	1.08E+02	1.32E+01	2.09E+05	1.86E+05	2.43E+07	7.94E+06	0.991

**Figure 3:**
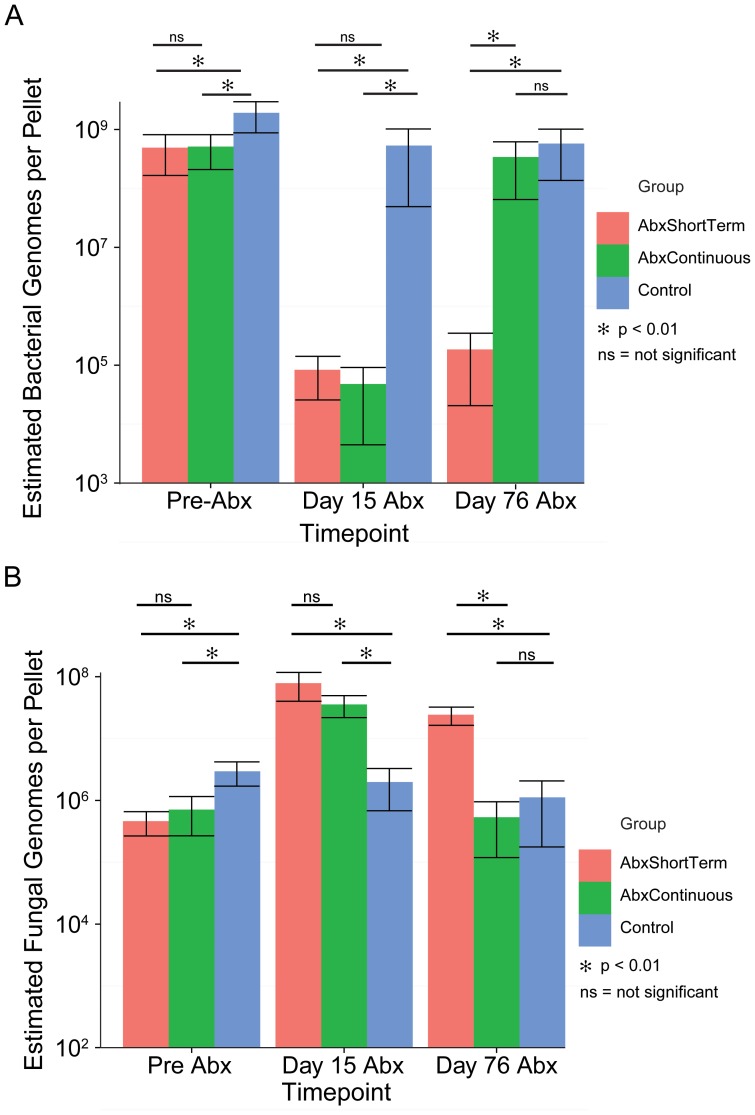
Numbers of organisms per stool pellet. Values from QPCR were corrected to yield an estimate of the true numbers of organisms by accounting for differential DNA yield, numbers of rRNA gene copies per genome, and efficiency of detection. A) Estimated numbers of bacterial genomes per pellet. Note that during antibiotic treatment (Day 15), most of the 16S rRNA gene copies were derived from food and do not correspond to intact organisms. (B) Estimated numbers of fungal genomes per pellet. The x-axis shows the time point studied, and the y-axis shows the inferred numbers of genomes. Each study group is indicated by the color code to the right of the figure panels.

Taking these factors into account, we found that in the absence of antibiotic treatment, a typical stool pellet contained 5×10^8^–2×10^9^ bacteria, and this dropped to ∼5×10^4^ bacteria after 15 days of antibiotic treatment (Figure 3A and Table 1). Below we show that 5×10^4^ is in fact an overestimate, because most of the 16S DNA was in fact derived from bacterial DNA in sterile mouse food. Bacteria returned to their former numbers after cessation of antibiotic treatment. Fungal genomes were much less abundant initially, only in the range of 5×10^5^–2×10^6^ per pellet (Figure 3B and Table 1). After 15 days of treatment with antibiotics, the numbers increased to ∼5×10^7^, or an increase of 25–50 fold. Fungal genome numbers remained high for the period of antibiotic treatment. Eight weeks after cessation of antibiotic treatment, counts in the ABXShortTerm groups returned to the pretreatment level. Thus changes in fungal cell abundance were substantial, though less than suggested by the analysis in Figure 2B, which was normalized to the total weight of DNA, because total DNA went down with antibiotic treatment.

### Analysis of Bacterial Lineages using 454/Roche Deep Sequencing

To assess the representation of microbial lineages present and changes with antibiotic therapy, we analyzed the longitudinal DNA samples using 454/Roche pyrosequencing. DNA was purified from stool from 13 time points (Figure 1). Bacterial sequences were amplified using primers matching the 16S rRNA gene V1V2 region [36], [42]. Sequencing yielded 239,867 reads, which were condensed into OTUs at 97% similarity and taxonomy assigned using the RDP classifier [43].

**Figure 4:**
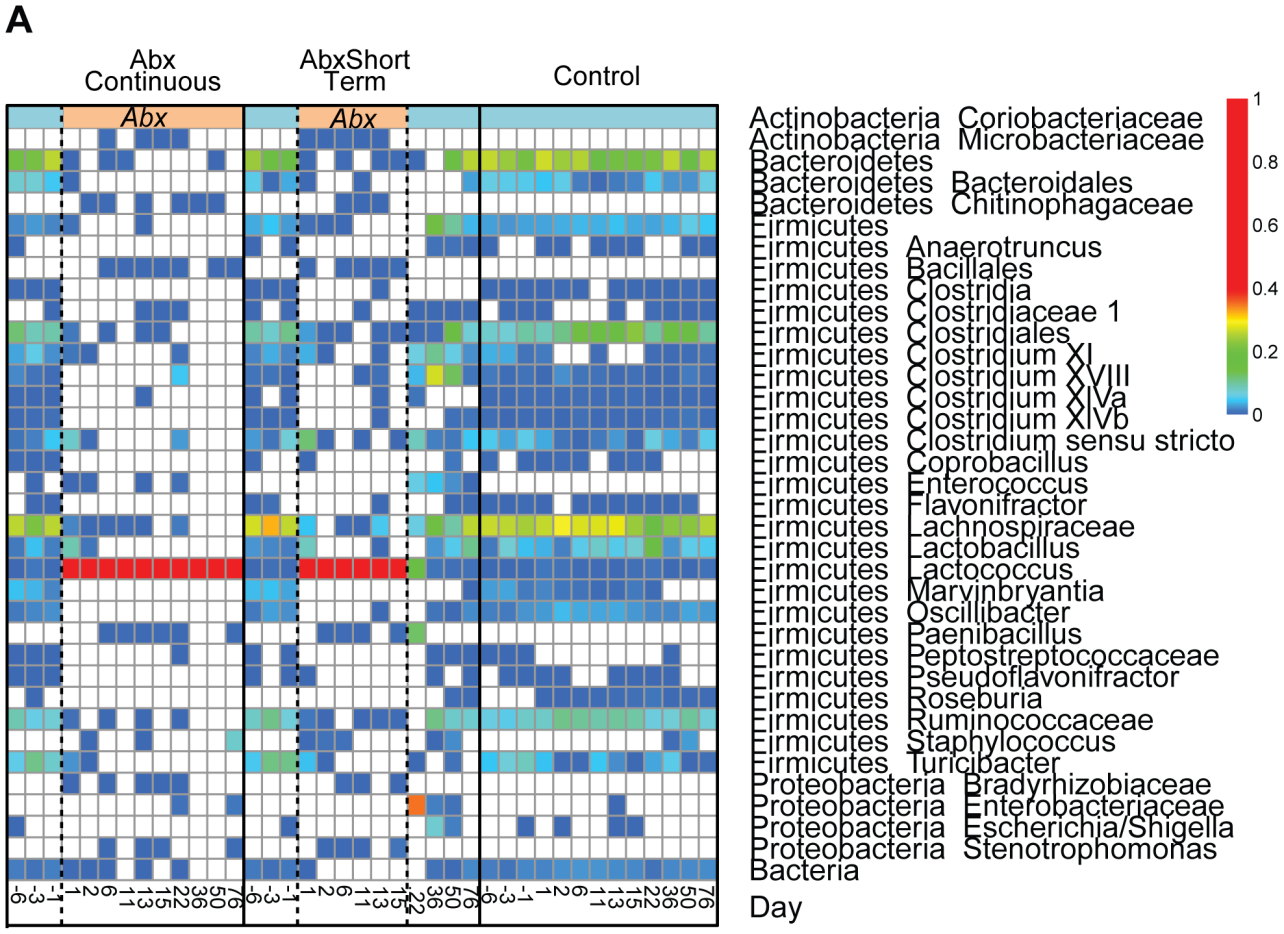
Longitudinal analysis of bacterial lineages inferred from 16S rRNA gene sequencing. Bacterial lineages detected are summarized in heat map format. Each column shows the average for the ten mice in each group and at the time point indicated. Sequence samples were rarefied to a standard 200 reads per individual before averaging. The periods of antibiotic treatment are indicated at the top in salmon color, the periods off antibiotic by light blue. The day of treatment is indicated at the bottom. The color code to the right indicates the proportions.

Prior to antibiotic treatment, communities were dominated by the Firmicute lineage *Lachnospiraceae* and the Bacteriodetes lineage *Bacteroidales,* along with a substantial number of less abundant lineages (Figure 4; Figure S2 A-I presents time points for each mouse individually). After one day of antibiotic treatment, the previously dominant lineages decreased sharply in abundance, and *Lactococcus* became the dominant community member. At later times under antibiotic treatment *Lactococcus* was the predominant or sole lineage detectable.

Five aliquots of sterile mouse chow were analyzed by amplification with the V1V2 primers and 454/Roche pyrosequencing, revealing that a single *Lactococcus* OTU was the predominant phylotype in both stool from antibiotic treated animals and in chow (Figure S3). We thus conclude that *Lactococcus* DNA is present in sterile mouse food, and that the antibiotic treatment eliminated the great majority of live bacteria, i. e. the 5×10^4^ bacteria detected per pellet in Table 1 represents mostly bacterial DNA in food.

After antibiotic treatment was stopped for the ABXShortTerm group, major groups that were predominant before antibiotic treatment returned to their former levels, but at different rates. An OTU classified as *Lachnospiraceae* and several OTUs classified as *Clostridium* returned within one week. Several other clades, including *Ruminococcaceae* and other Firmicutes increased in proportion by two weeks after cessation of treatment. *Bacteroidales* did not fully return until the end of the experiment at eight weeks. *Enteroccocus*, *Escherichia*, and *Paenibacillus*, which were not dominant members of the communities in the Control or antibiotic treated groups, had elevated proportions over the recovery period but decreased in relative abundance after eight weeks off antibiotics. Table S1 presents a statistical analysis of the bacterial lineages detected and their behavior over the time course studied.

**Figure 5:**
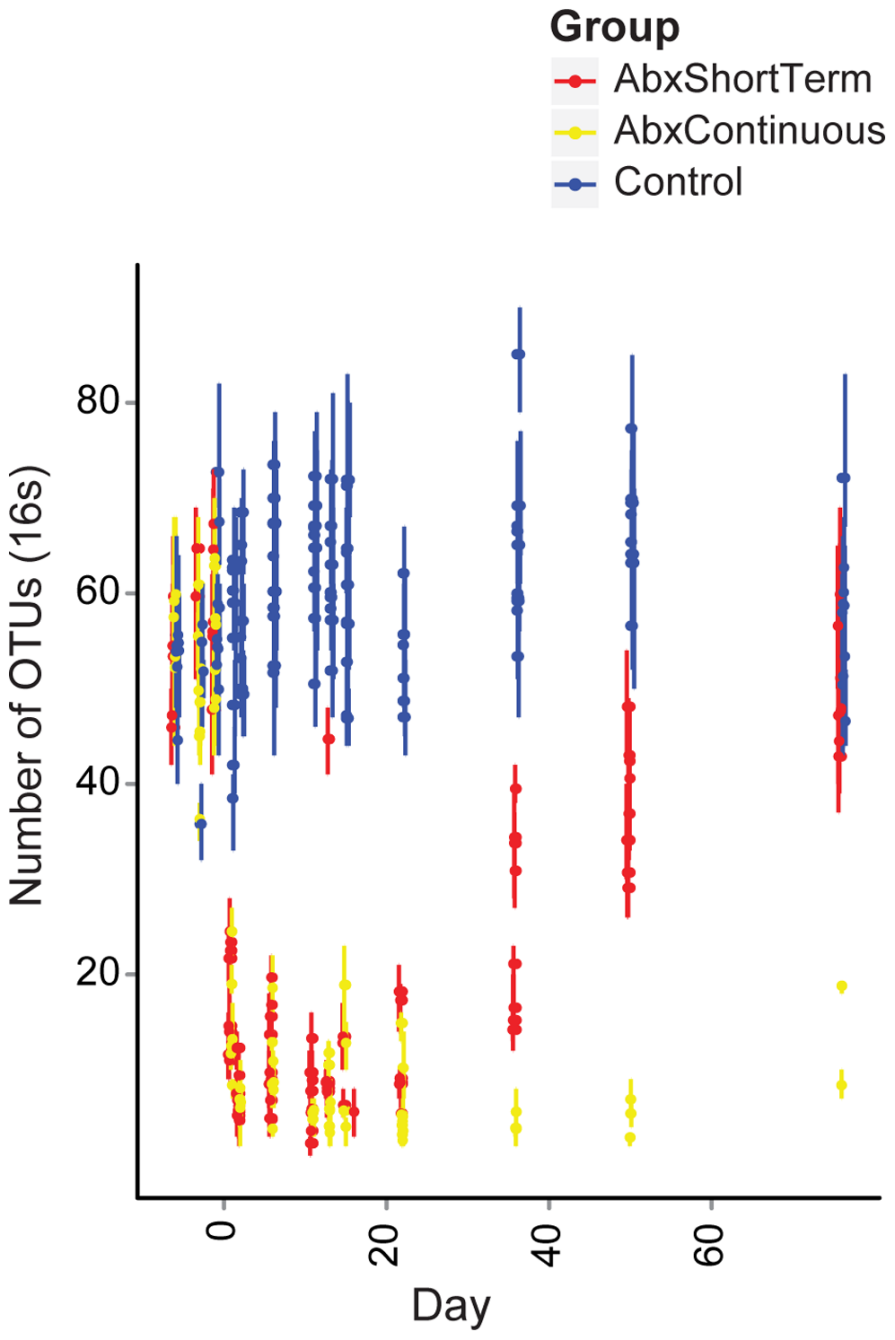
Abundance analysis of observed bacterial lineages. Each sequence set for each animal was rarefied to 200 sequences per sample 10 times, and the number of different OTUs assessed. Means are indicated by points, error bars indicate the range observed. The groups studied are indicated by the key at the right.

Changes in the types of bacterial lineages were paralleled by changes in the species richness (Figure 5). Prior to antibiotic treatment, 54.6 (SD = 6.9) phylotypes were detected after data from each mouse was normalized to 200 reads. After 2 days of antibiotic treatment, this fell to 7 (SD = 2.0) and persisted for the remainder of the antibiotic treatment (p<2.2×10^−16^ for comparison to the pretreatment group, Friedman test). Upon cessation of antibiotic treatment, the community slowly returned to its former richness reaching 49.4 (SD = 5.6) lineages over 61 days, still less than the corresponding Control group which averaged 57.2 (SD = 17) lineages on the same day (p = 0.02 Mann-Whitney U test).

### Analysis of Microeukaryotes using 454/Roche Deep Sequencing

To characterize microeukaryotes, we sequenced selected samples using 18S and ITS amplicons. To compare samples from the different treatment groups, 134,677 ITS sequences and 26,355 18S sequences were generated, OTUs were formed, and taxonomic attribution was preformed with BROCC [44]. The 18S amplicon is more universal than the ITS amplicon [44], while the ITS amplicon provides greater resolution for some fungal lineages [45], so both were used [37]. To check that the two amplicons were yielding consistent information, we compared sequence samples from 15 mice amplified using both amplicons. Sequence samples were characterized by generating pairwise UniFrac distances, then the distance matrices for each were compared using Procrustes analysis. This showed high correlation between the two (p<0.0001, no better fits after 10^4^ permutations) and compositional comparison also showed similar profiles (Figure S1).

**Figure 6:**
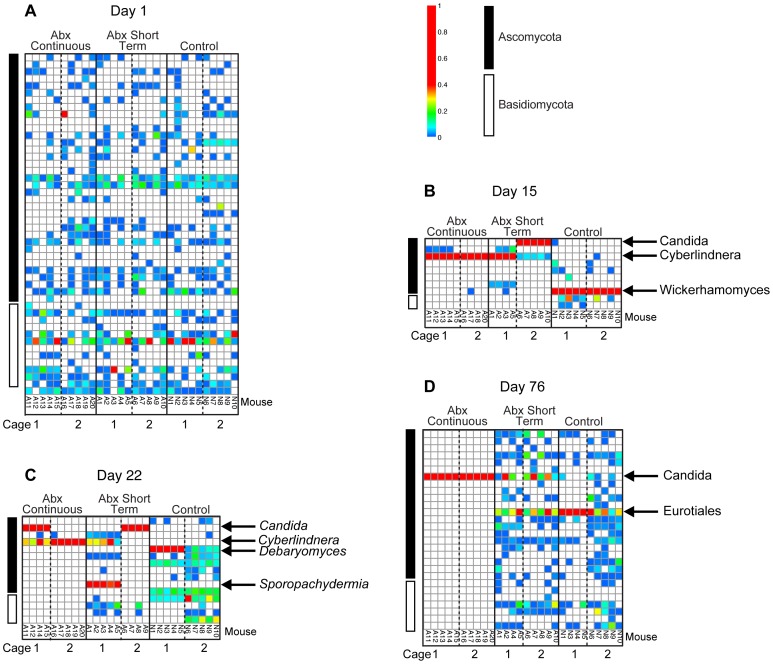
Longitudinal analysis of fungal lineages inferred from ITS rRNA gene sequencing. Fungal lineages detected are summarized in heat map format. Data for four days are shown (days 1, 15, 22, and 76). Each column indicates a single mouse. The groups tested are indicated at the top of the columns. The ten mice in each of the three treatment groups were each housed in two cages of five each. The distribution of mice in cages is indicated at the bottom of the columns. The day of treatment is indicated at the top. The color codes at the top right indicate the proportions and Phyla of origin.

The longitudinal behavior of fungal communities was explored in detail using the ITS amplicon, which revealed strong effects of both antibiotic treatment and caging history of the animals (Figure 6; the full set of time points, with each animal shown individually, is in Figure S4A-I). In the control animals (five mice in each of two cages), although the composition of the bacterial community remained relatively stable, the fungal community changed dramatically. For four samples taken over the first eight days, most of the ten mice in the two cages showed colonization by diverse fungal lineages, and no lineage predominated. By day 15, however, the situation had changed radically, with both cages dominated by a phylotype annotated as *Wickerhamomyces*. This changed by day 22, with cage 1 dominated by *Debaryomyces*, and the second cage showing more diverse colonization, where *Debaryomyces* was present but not predominant. The outgrowth of *Debaryomyces* in cage 1 correlated with the increase in abundance of total DNA in the 18S QPCR analysis in the control animals at day 22 (Figure 3), which also occurred only in cage 1. By day 76 the controls had changed again. At this time both cages were dominated by *Eurotiales*, though the abundance was greater in cage 1 than cage 2. These findings document radical changes in murine gut fungi 1) in a single mouse facility 2) for mice on a homogeneous diet, 3) in the absence of any intervention, and 4) differing between cages.

For the antibiotic treated animals, the communities were diverse prior to initiation of treatment, then under treatment showed cage-specific patterns of divergence. By day 2, *Clavispora* and *Cyberlindnera* were predominant in both cages in the ABXContinuous group and cage 1 in the ABXShortTerm group. In cage 2 in the short term group the mice were more heterogenous, with *Candida*, *Cyberlindnera*, and *Pichia* dominating in different mice. By day 6 this had changed, with *Cyberlinderna* dominating in three cages (both ABXContinuous and ABXShortTerm cage 1), and *Candida* dominating in all mice in ABXShortTerm cage 2. This pattern persisted at day 15, but by day 22 one ABXContinuous cage was dominated by *Candida*, and by day 76 *Candida* was the only fungus detectable in both ABXContinuous cages. For the ABXShortTerm mice, *Sporopachydermis* dominated in cage 1 and *Candida* in cage 2 on day 22, then the fungal populations returned to a more diverse mixture by day 76, but *Candida* was relatively abundant in most of the short term treated mice at the last time point studied. Thus these data emphasize the heterogeneity of the community responses in individual cages, but also the robust persistence of the *Candida* community. Table S2 presents a statistical analysis of the fungal lineages detected and their behavior over the time course studied. A few samples were also analyzed with the 18S rRNA gene amplicon and generally yielded similar results (Figure S1). An analysis of mouse chow DNA using the ITS amplicon showed no obvious relationship to the major lineages detected in pellets (Figure S3).

**Figure 7:**
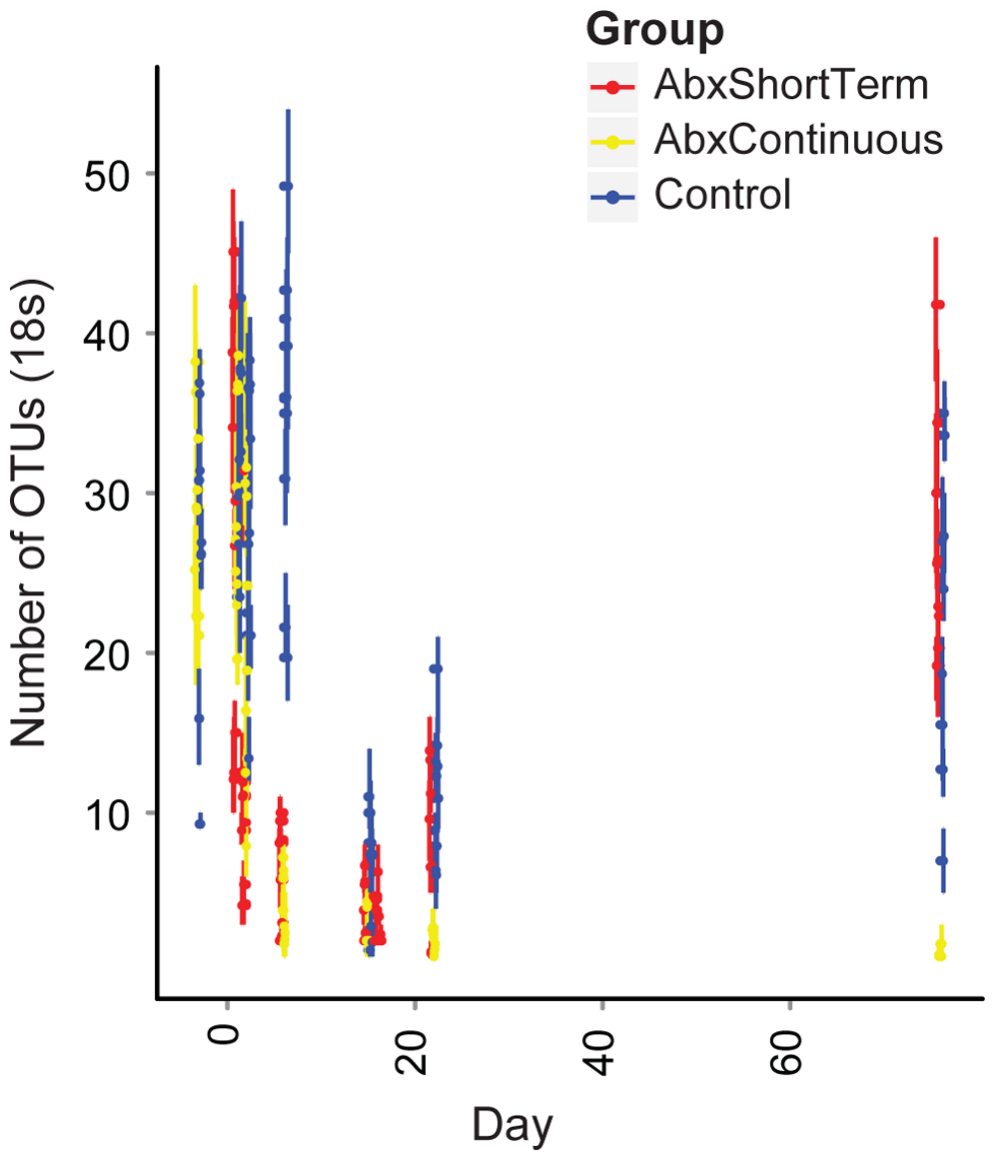
Abundance analysis of observed fungal lineages. Each sequence set was rarefied to 200 sequences per sample, and the number of different OTUs assessed. Means are indicated by points, error bars indicate the range observed. The groups studied are indicated by the key at the right.

Initially an average of 30.7 (SD = 9.9) phylotypes were detected in the ITS data per animal (Figure 7), but these numbers fell to 5.2 (SD = 2.9) per animal during antibiotic treatment as the *Candida* overgrew the community (p = 8.3×10^−12^, Friedman test). Upon cessation of antibiotic treatment the number of phylotypes returned to their former levels (31.1, SD = 8.0, p = 0.673, Mann-Whintey U). Thus the fungal community returned to its original richness, but despite this, the contribution of *Candida* was higher than before treatment.

### Community Comparisons using Unifrac

To compare community structures over the course of antibiotic treatment, the 454 data for the 16S and ITS tags were analyzed using weighted [46] and unweighted [47] Unifrac (Figure S5). We tested for differenced in community composition between the control group and the treatment groups at each time point by comparing distances within the control group to distances between control and treatment groups. Significance was determined using the Mann-Whitney test.

The ABXShortTerm and ABXContinuous groups were not significantly different from the Control group before antibiotic treatment for either the bacterial or fungal communities (Figure S5). After one day of treatment, both groups were significantly different from the Control in both weighted and unweighted Unifrac and remained distinct throughout the antibiotic treatment (Figure S5). One week after the ShortTermABX group stopped receiving antibiotics, both the bacterial and fungal communities remained significantly different from the other two (Figure S5). On day 76, 61 days after antibiotic cessation, differences between the bacterial communities were slight but still achieved significance in the weighted and unweighted analysis, and some but not all comparisons were significant for the ITS analysis. Thus by the last time point the communities had approached but not completely returned to their pre-treatment states (p<0.005).

## Discussion

**Figure 8:**
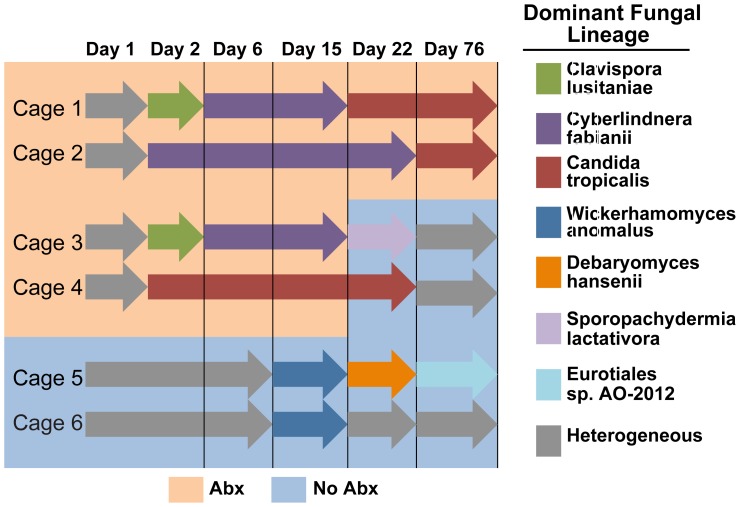
Longitudinal variation in fungal abundance in the six cages studied. Each cage is shown as a row labeled at left, the types of fungi detected are shown at the right. The background shading indicates the presence or absence of antibiotics (key at bottom).

We report a longitudinal study of the bacterial and fungal communities in the mouse gut after treatment with a cocktail of four antibiotics. Our main observations were that 1) bacterial communities dropped sharply in abundance, then recovered to near to their starting state after cessation of antibiotic treatment, 2) fungal communities increased in abundance with the fall in bacterial abundance, and 3) waves of fungal colonization swept through all of the cages in our study, including the untreated controls (Figure 8). We discuss each of these findings in turn below.

Multiple studies have examined the effects of antibiotic treatment on the gut microbiome in vertebrates. Dethlefsen et al. studied the gut microbiome of three humans treated with ciprofloxacin [48], and found that while diversity was reduced upon treatment, the response of bacterial lineages differed among the individuals studied. Communities mostly returned to their initial states with cessation of treatment, though detectable differences remained. Hill et al. studied mice subjected to an extreme antibiotic treatment (ampicillin, gentamicin, metronidazole, neomycin, and vancomycin) designed to eradicate gut bacteria [26], and found that gut could be cleared out to such an extent that only food sequences were detectable, paralleling findings here. A profound and reproducible change was observed in the composition of the microbiota at both luminal and tissue associated sites. Antonopoulos et al. studied mice exposed to a cefoperazone regimen followed by a 6 week recovery period [49]. They observed bacterial counts drop by 3 orders of magnitude during antibiotic treatment and saw significant compositional changes and decreased diversity in bacterial communities after cessation of antibiotics. Peterfreund et al. studied bacteria and fungi of the hamster gut following treatment with clindamycin and C. difficile infection [29], and found that after treating C. difficile with an antitoxin antibody the community reached an altogether new stable state.

The bacterial communities studied here were initially dominated by *Bacteriodetes* and *Lachnospiraceae*,but changed quickly upon initiation of antibiotic treatment, reaching a stable state by day two. At this time, the number of inferred organisms had dropped >3 orders of magnitude, to the point that the 16S rRNA gene sequences present in stool were mostly transients from food, documenting near complete clearance of bacteria from the gut. Upon cessation of antibiotic treatment, the bacteria returned to nearly their original state, though perturbations remained. Taken together with previous studies, these findings emphasize that of the response of gut bacteria to antibiotics vary quite substantially with the choices of host organism, antibiotic regimen, and other experimental features.

The bacterial populations in the control animals remained relatively stable over the time course, dominated by *Bacteriodetes* and *Lachnospiraceae* throughout. A study by Schloss et al. showed that the bacteria of the gut of new borne pups varied over time initially, but stabilizes later in life [50], paralleling studies of human infants [51]. Our data showed that the animals studied here had indeed achieved a stable adult bacterial profile, allowing us to attribute changes to antibiotic treatment. However, the same was not true for fungi.

Fungal communities changed radically over the time course studied in association with the caging history of the mice. The amounts of fungi detected here prior to Abx treatment were comparable to previous studies. Qin et al. [52] suggested that the proportion of eukaryotic DNA in human stool was <0.1%, while FISH analysis of murine gut biofilms [53] suggested 0–10% fungi, with a median of 2%. Our data in the absence of Abx suggested fungal proportions in the range of 0.1–0.3%.

Unexpectedly, even the Control group showed waves of succession that differed in each cage (Figure 8). Cage 1 was first heterogenous, then dominated by *Wickerhamomyces*, then *Debaryomyces*, then *Eurotiales*. Cage 2 was heterogenous through the first six days, then dominated by *Wickerhamomyces*, then again heterogeneous. These data document a quite surprising degree of fungal variation in healthy laboratory mice. Previous studies have shown that colonization by specific bacteria in mice can strongly influence the outcome of immunological assays [54]. Given the recently reported importance of fungi in mouse models of IBD [31], our data suggest that researchers will need to take care to monitor and control fungal populations to obtain meaningful data. The mice in this study were housed on a conventional SPF environment. It would be useful to compare housing in a barrier facility where food, water, and bedding have all been sterilized. Colitis phenotypes in genetically-determined mouse models are known to vary in different facilities, suggesting that it will be useful to assess the role of variability in fungal colonization.

In the presence of antibiotics, the fungal community showed several waves of succession, which again differed between cages. During Abx treatment, the fungal proportions exceeded 99% of all microbes. Depending on the cage and time point, the communities could be heterogeneous, or dominated by *Clavispora*, *Cyberlindnera*, *Sporopachydermia* or *Candida*. In cases where communities in a cage were dominated by a single fungal lineage, this was true of all mice in the cage at that time point. One likely explanation is that coprophagia resulted in all mice in a single cage acquiring the same fungal colonists. Some of these fungal lineages were seen sporadically in contamination controls (Figure S6), suggesting that gut fungi may have been acquired episodically from the environment.

*Candida* was a particularly robust colonist in the presence of antibiotics. By day 76, all the ABXContinuous mice were colonized at a high level exclusively by *Candida*. Further supporting the robustness of *Candida*, results from cage two in the ABXShortTerm group by chance provides a competition experiment. Of the five mice analyzed on day two, two were colonized with *Candida*, one with *Cyberlindnera*, one with *Pichia*, and one with both *Candida* and *Cyberlindnera* (Figure S4, part C). Coprophagia would presumably allow the three species to compete for colonization opportunities. By day six, all mice were colonized with *Candida*, and this persisted through the cessation of antibiotic treatment by day 22. At the end of the experiment on day 76, *Candida* was still more abundant in the ABXShortTerm group than prior to treatment, all emphasizing that *Candida* was favored by the antibiotic treatment and persisted subsequently. These data motivate more careful studies of fungal blooms, and particularly Candida, in human subjects undergoing antibiotic treatment.

## Methods

### Mouse Husbandry

Thirty C57B6 eight week old female mice were purchased from Jackson Laboratory and placed on a standardized diet for two weeks prior to the study to stabilize their flora. Mice were housed by treatment group with five mice per cage. Mice treated with antibiotics received ampicillin, neomycin, vancomycin, and metronidazole in water. Water was spiked with aspartame in both the treated groups and controls. Mice were fed AIN-76A Rodent Diet from Research Diet (D10001) for the course of the study, which includes 15% casein lactic. The Institutional Review Board of the University of Pennsylvania approved all IACUC protocols (protocol #803408). The animal care facility is operated by the University Laboratory Animal Resources, which is fully accredited by the American Association for Accreditation of Laboratory Animal Care. Laboratory animals are maintained in accordance with the applicable portions of the Animal Welfare Act and their guidelines prescribed in the DHHS publication, “Guide for the Care and Use of Laboratory Animals”. Mice are euthanized using C02 inhalation. Methods for euthanasia are consistent with the recommendations of the Panel on Euthanasia of the American Veterinary Medical Association.

### DNA Extraction

DNA was extracted from 1–2 mouse pellets per mouse per time point. Samples were homogenized for 80 seconds on a Mini-BeadBeater-16 (BioSpec) in Lysing Matrix E tubes (MP Biomedical). Samples were then incubated at 95°C for 15 minutes and then cooled on ice for 60 seconds. Then samples were extracted with the PSP DNA extraction kit and using a protocol described previously [44]. Sequences of oligonucleotides used in this study are presented in Table S3.

### DNA Sequencing and QPCR

Extracted DNA was quantified with the Picogreen system. Typical volumes produced, used in the calculations in Table 1, were 250 microliters. DNA was amplified using primers annealing to the V1V2 region of the 16S bacterial gene or the ITS1 fungal rRNA gene spacer, and amplified with AccuPrime taq with Buffer 2 (Invitrogen). Thermocycler protocols for 16S [36] and ITS and 18S [44] amplicons were described previously. PCR amplicons were purified with Agencourt AMPure XP beads. Sequencing was performed on a 454 Junior using Titanium chemistry. For both 16S and ITS amplicons, DNA free water was subjected to the same purification procedure and analyzed by 454/Roche pyrosequencing (Figure S6). A subset of samples showed recoverable sequences, but stool samples showed distinct community composition, so we conclude that environmental contamination made a minimal contribution to the samples analyzed.

16S qPCR was performed using the Taqman method as described previously [26]. The Taqman method was chosen because it provides good specificity for bacterial sequences in complex mixtures, but reconstruction experiments show that the Taqman values were on average 11-fold lower than for parallel Syber Green assays, and follow up studies suggested the Syber Green values were reliable. Thus a correction was added for the quantitative comparison in Figure 3A and Table 1. 18S qPCR was performed with the Applied Biosystems SYBR Green Fast chemistry. Ten µL SYBRGreen FAST 2×master mix, 1 µL of each primer diluted to 20 pM, and 8 µL DNA were added to each reaction. Thermocycling was performed as follows: samples were initially denatured once for 2 minutes at 50°C then 10 minutes at 95°C. Then samples were cycled 40 times with a dissociation step at 95°C for 15 seconds and an annealing and extension step at 60°C for 1 minute. Primer sequences for these assays can be found in Table S3. All DNA sequences generated in this study have been submitted to the Sequence Read Archive under accession number SRP020503.

### Bioinformatic Analysis

Bioinformatic analysis was performed with the QIIME software package [55] using default parameters except where indicated, and using R. Fungal reads were queried against the nt database using NCBI’s blastn tool and then classified with the BROCC classifier [44].

Eukaryotic PCoA analysis was performed using taxonomic relationship corresponding to the NCBI Taxonomy [56]. Because de novo tree construction using the ITS1 region is not feasible due to length variation inherent in the ITS gene [44], [57]–[59], we chose to asses Unifrac distances between eukaryotic communities using the NCBI Taxonomy to generate taxonomic trees. To transform the taxonomy into a phylogenetic tree, all edges between taxa were assigned equal weight. Classifications were curated manually for parsimony. While the fungal taxonomy is imperfect and in a state flux [45], [60]–[62], we note that in practice the Unifrac metric is relatively robust to the method used in creating phylogenetic trees [63]. Comprehensive data on OTU mean proportions and counts are in Tables S4–S7. Statistical significance for treatment groups was determined using the nonparametric Mann-Whitney test in R. Permanova tests and Procrustes analysis were performed in QIIME.

## Supporting Information

Figure S1 **Comparison of microeukaryote lineages specified by the ITS and 18S amplicons.** A) Heat maps comparing selected samples analyzed using both the 18S and ITS amplicons. Each column shows the average for mice in the group and at the time point indicated rarefied to 200 reads per individual. The color code to the right indicates the scale. B) Procrustes analysis comparing results for the 18S and ITS analysis. Data from the 18S and ITS amplicons for each mouse are shown by balls connected by a line.
(TIF)

Figure S2 **Heat maps showing the composition of bacterial communities inferred from 16S sequence data for each time point, with each mouse shown individually.** The scale of relative proportions is shown on the far right.
(TIF)

Figure S3 **16S and ITS sequences recovered from five samples of mouse chow.** A) Sequences from the 16S analysis. B) Sequences from the ITS analysis. The scale of relative proportions is shown on the far right.
(TIF)

Figure S4 **Heat maps showing the composition of fungal communities inferred from ITS sequence data for each time point, with each mouse shown individually.** The scale of relative proportions is shown on the far right.
(TIF)

Figure S5 **PCoA analysis distances measures for bacterial (16S sequence data) and fungal (ITS sequence data) communities.** Distances matrices were calculated using weighted or unweighted UniFrac, then the pairwise distances between a treatment group and the control group compared to the distances within the control group on that day. Asterisks above each box and whisker plot indicate whether the comparison was significantly different.
(TIF)

Figure S6 **Comparison of contamination controls to experimental samples for the 16S A) and ITS B) amplicons.** “Extraction control” indicates sequences derived from blank purifications using DNA-free water. Each column showing mouse data is an average over all reads at that time point.
(TIF)

Table S1 **Bacterial lineages detected in this study and statistical analysis of longitudinal changes.** Each time course was separated into intervals, and species abundance was compared between the time intervals indicated in the column headings. Mean values were compared for each lineage using the Friedman test (non-parametric with repeated measures). P-values that survived Bonferroni correction are highlighted and bolded. Intervals were compared as follows: -Baseline: Comparison between groups among baseline time points. -Early ABX vs Control: The first two abx timepoints samples are compared to Control samples from the same time point. -Late ABX vs Control: All time points after the first two abx days when ABXShortTerm are still receiving abx. Control and abx treated samples are compared. -Recovery vs Control: All time points after abx were stopped for the ABXShortTerm group. Control and ABXShortTerm are compared. -Last Day Recovery vs Control: The last day of the study only. Control and ABXShortTerm are compared. -Early ABX vs Late ABX: The first two abx time points are compared to later (days 6–15) abx time points. ABXShorterm and ABXContinuous are lumped in together.
(XLSX)

Table S2 **Fungal lineages detected in this study and statistical analysis of longitudinal changes.** Each time course was separated into intervals, and species abundance was compared between the time intervals indicated in the column headings. Mean values were compared for each lineage using the Friedman test (non-parametric with repeated measures). p-values that survived Bonferroni correction are highlighted and bolded. Comparisons where a species did not appear in both time periods or groups being compared are marked n/a. Intervals were compared as follows: -Baseline: Comparison between groups among baseline time points. -Early ABX vs Control: The first two abx timepoints samples are compared to Control samples from the same time point. -Late ABX vs Control: All time points after the first two abx days when ABXShortTerm are still receiving abx. Control and abx treated samples are compared. -Recovery vs Control: All time points after abx were stopped for the ABXShortTerm group. Control and ABXShortTerm are compared. -Last Day Recovery vs Control: The last day of the study only. Control and ABXShortTerm are compared. -Early ABX vs Late ABX: The first two abx time points are compared to later (days 6–15) abx time points. ABXShorterm and ABXContinuous are lumped in together.
(XLSX)

Table S3 **Oligonucleotides used in this study.**
(XLSX)

Table S4 **Means, variances, and standard deviations for bacterial OTUs within treatment groups and time points rarefied to 200 reads per sample.**
(XLSX)

Table S5 **Means, variances, and standard deviations for fungal OTUs within treatment groups and time points rarefied to 200 reads per sample.**
(XLSX)

Table S6 **OTU table of bactieral 16S sequences rarefied to 200 reads per sample.**
(XLSX)

Table S7 **OTU table of fungal ITS sequences rarefied to 200 reads per sample.**
(XLSX)
