# Supplementary figures and images for: Fungi of the Murine Gut: Episodic Variation and Proliferation during Antibiotic Treatment
Source: PLoS One. 2013 Aug 19;8(8):e71806. doi: 10.1371/journal.pone.0071806 (PMC3747063; doi:10.1371/journal.pone.0071806)

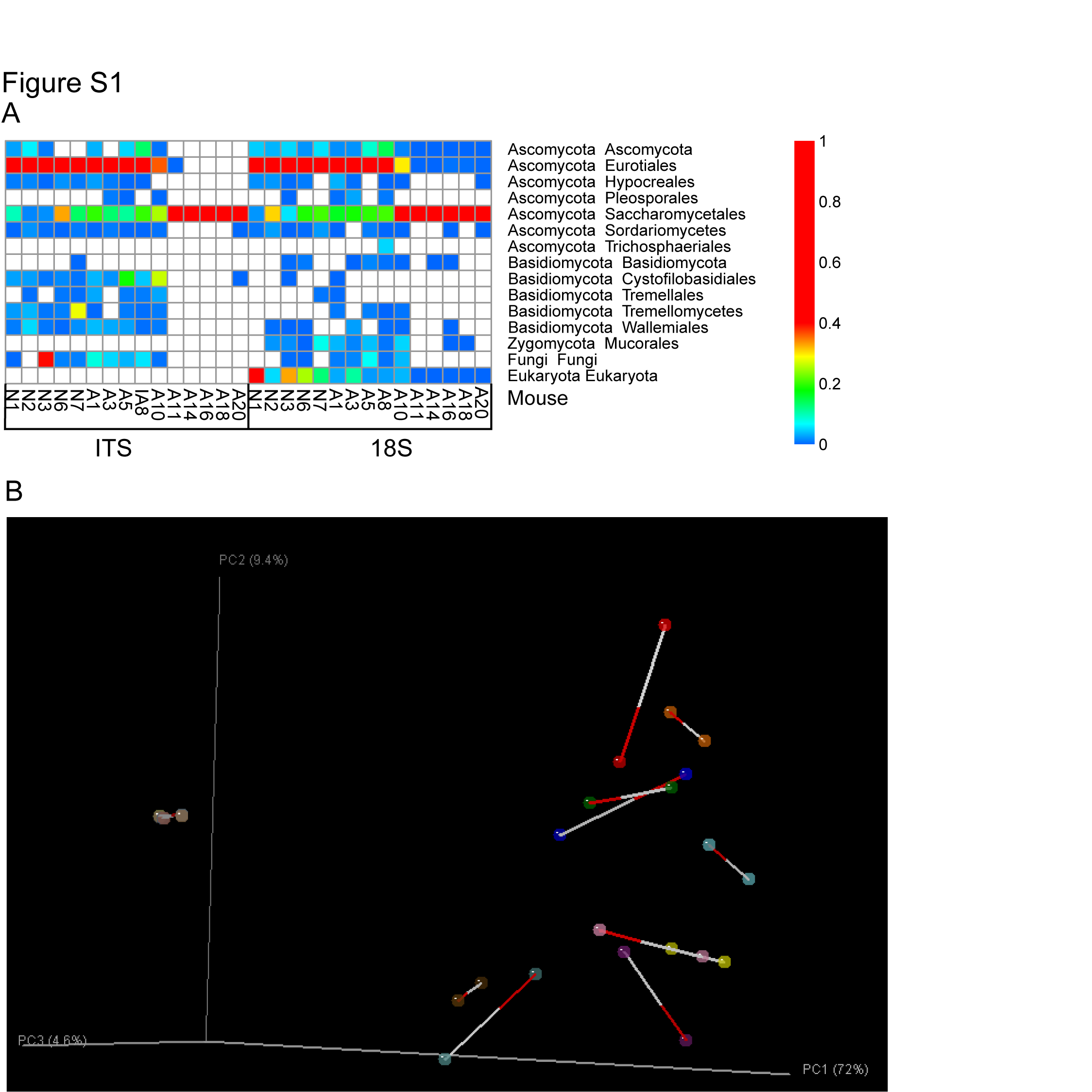

Supplement: Figure S1 — Comparison of microeukaryote lineages specified by the ITS and 18S amplicons. A) Heat maps comparing selected samples analyzed using both the 18S and ITS amplicons. Each column shows the average for mice in the group and at the time point indicated rarefied to 200 reads per individual. The color code to the right indicates the scale. B) Procrustes analysis comparing results for the 18S and ITS analysis. Data from the 18S and ITS amplicons for each mouse are shown by balls connected by a line. (TIF) [file pone.0071806.s001.tif]

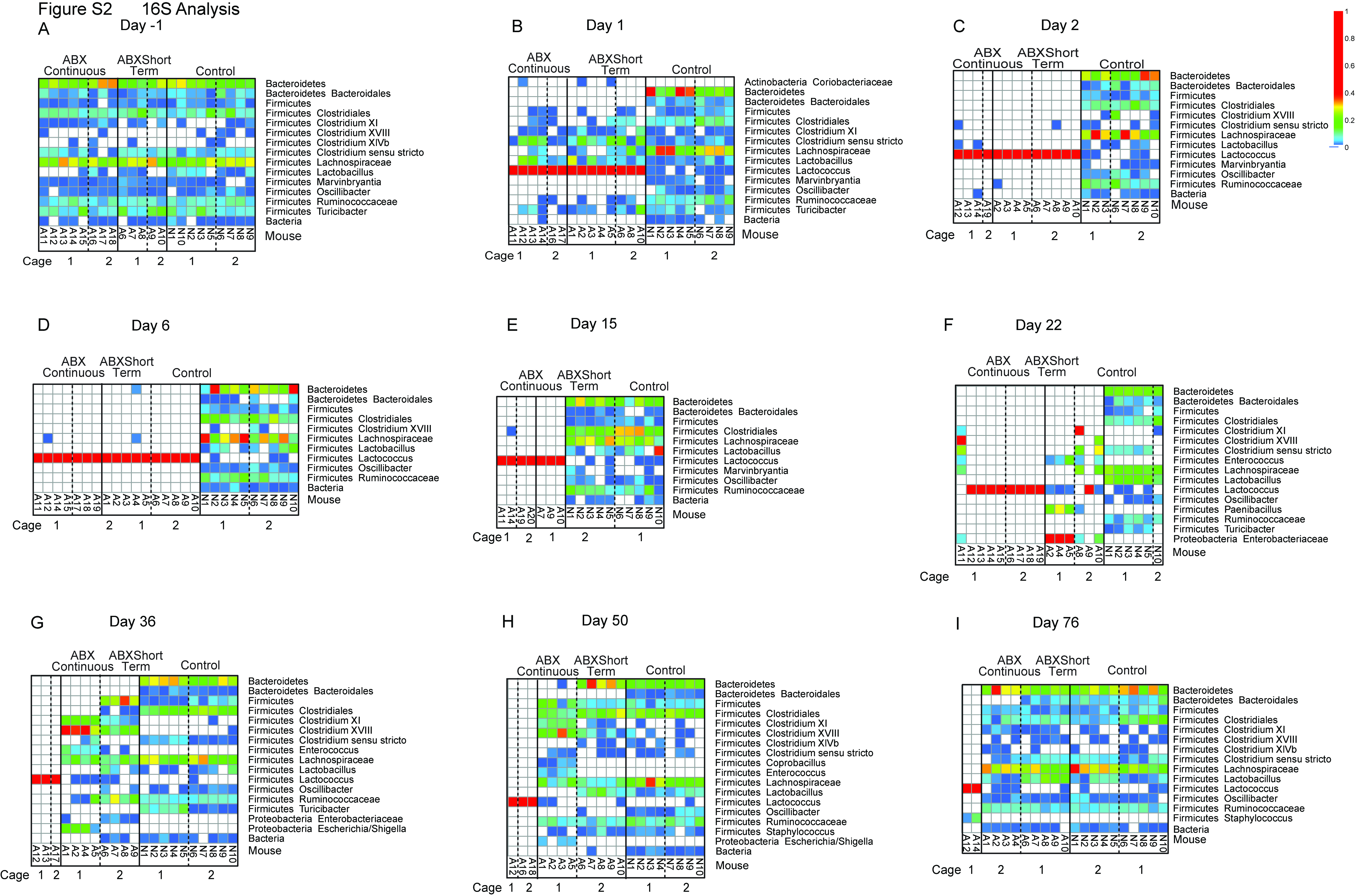

Supplement: Figure S2 — Heat maps showing the composition of bacterial communities inferred from 16S sequence data for each time point, with each mouse shown individually. The scale of relative proportions is shown on the far right. (TIF) [file pone.0071806.s002.tif]

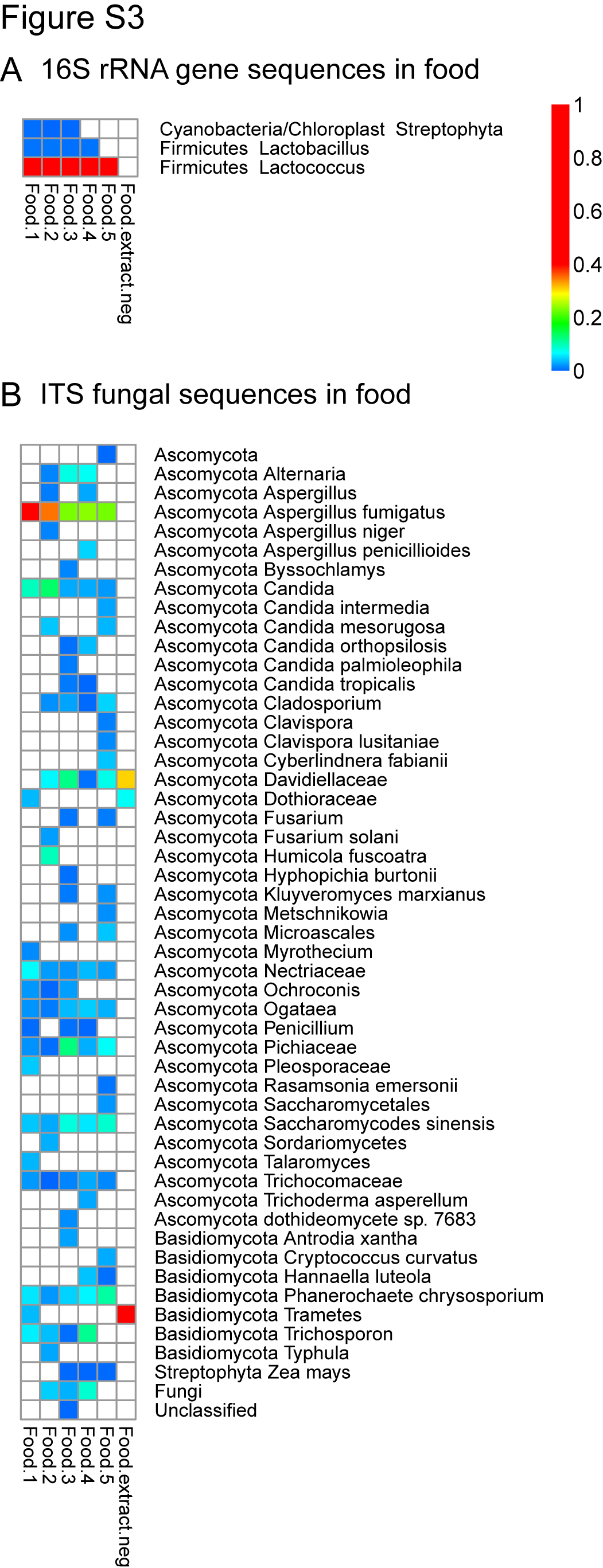

Supplement: Figure S3 — 16S and ITS sequences recovered from five samples of mouse chow. A) Sequences from the 16S analysis. B) Sequences from the ITS analysis. The scale of relative proportions is shown on the far right. (TIF) [file pone.0071806.s003.tif]

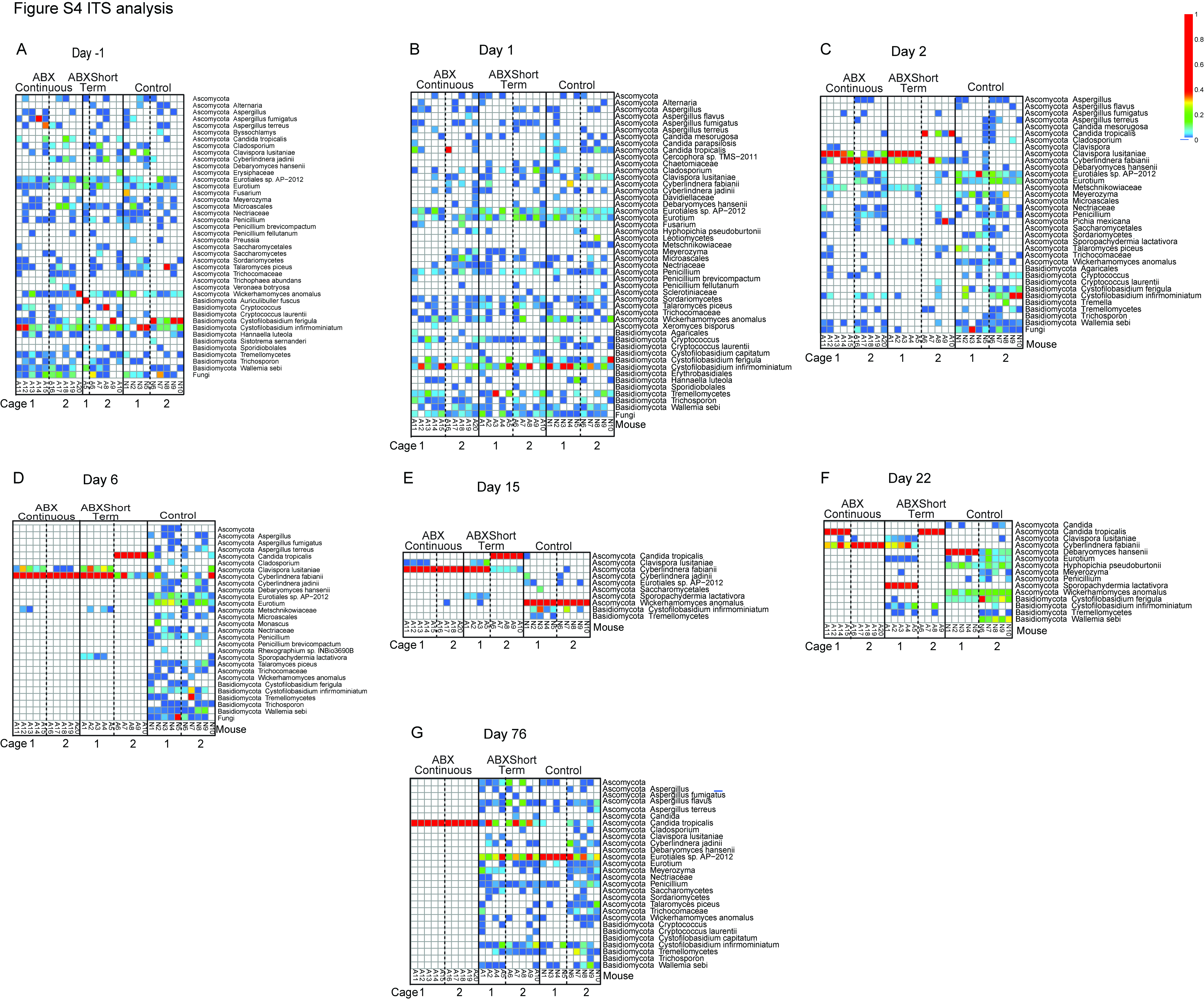

Supplement: Figure S4 — Heat maps showing the composition of fungal communities inferred from ITS sequence data for each time point, with each mouse shown individually. The scale of relative proportions is shown on the far right. (TIF) [file pone.0071806.s004.tif]

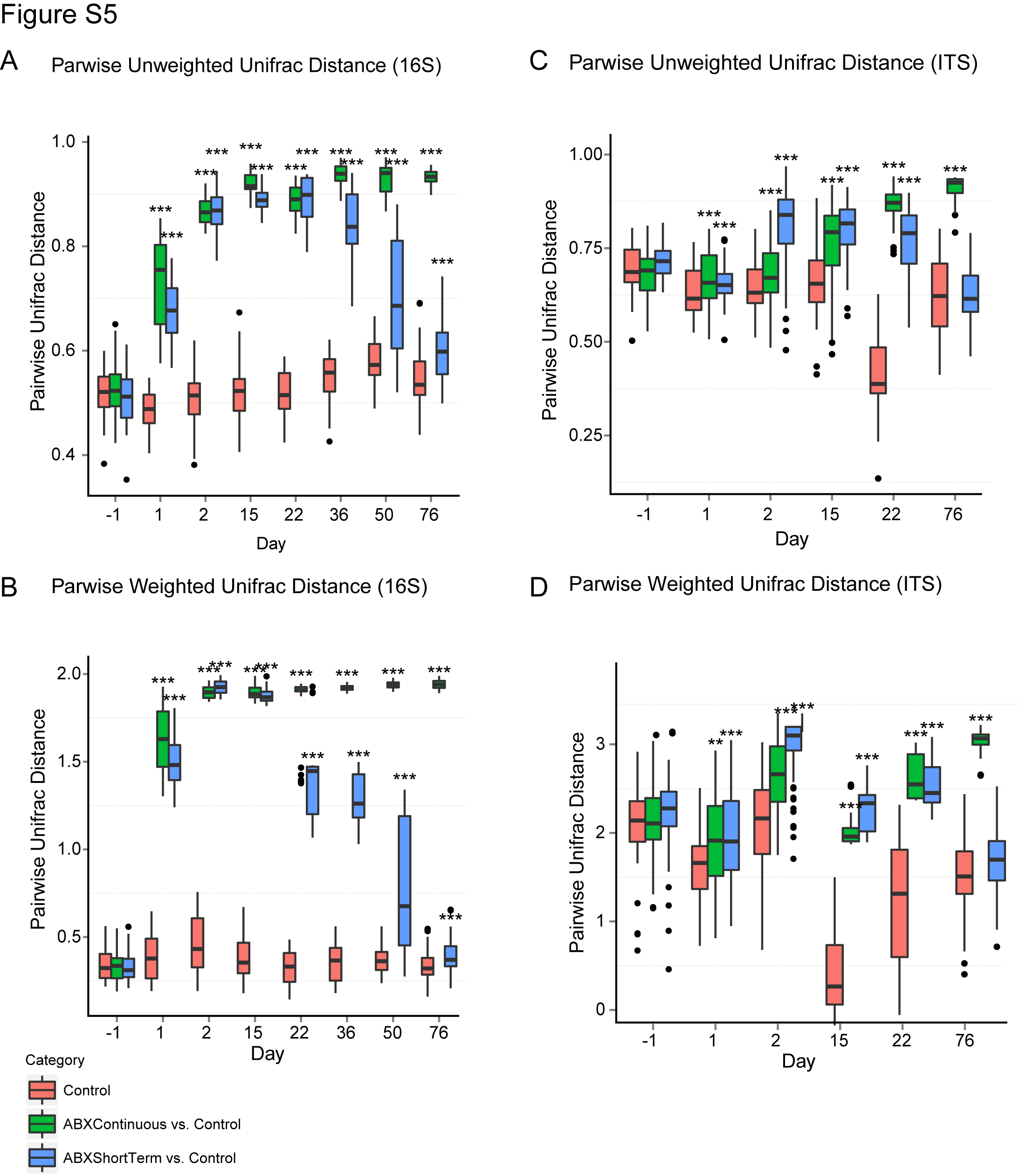

Supplement: Figure S5 — PCoA analysis distances measures for bacterial (16S sequence data) and fungal (ITS sequence data) communities. Distances matrices were calculated using weighted or unweighted UniFrac, then the pairwise distances between a treatment group and the control group compared to the distances within the control group on that day. Asterisks above each box and whisker plot indicate whether the comparison was significantly different. (TIF) [file pone.0071806.s005.tif]

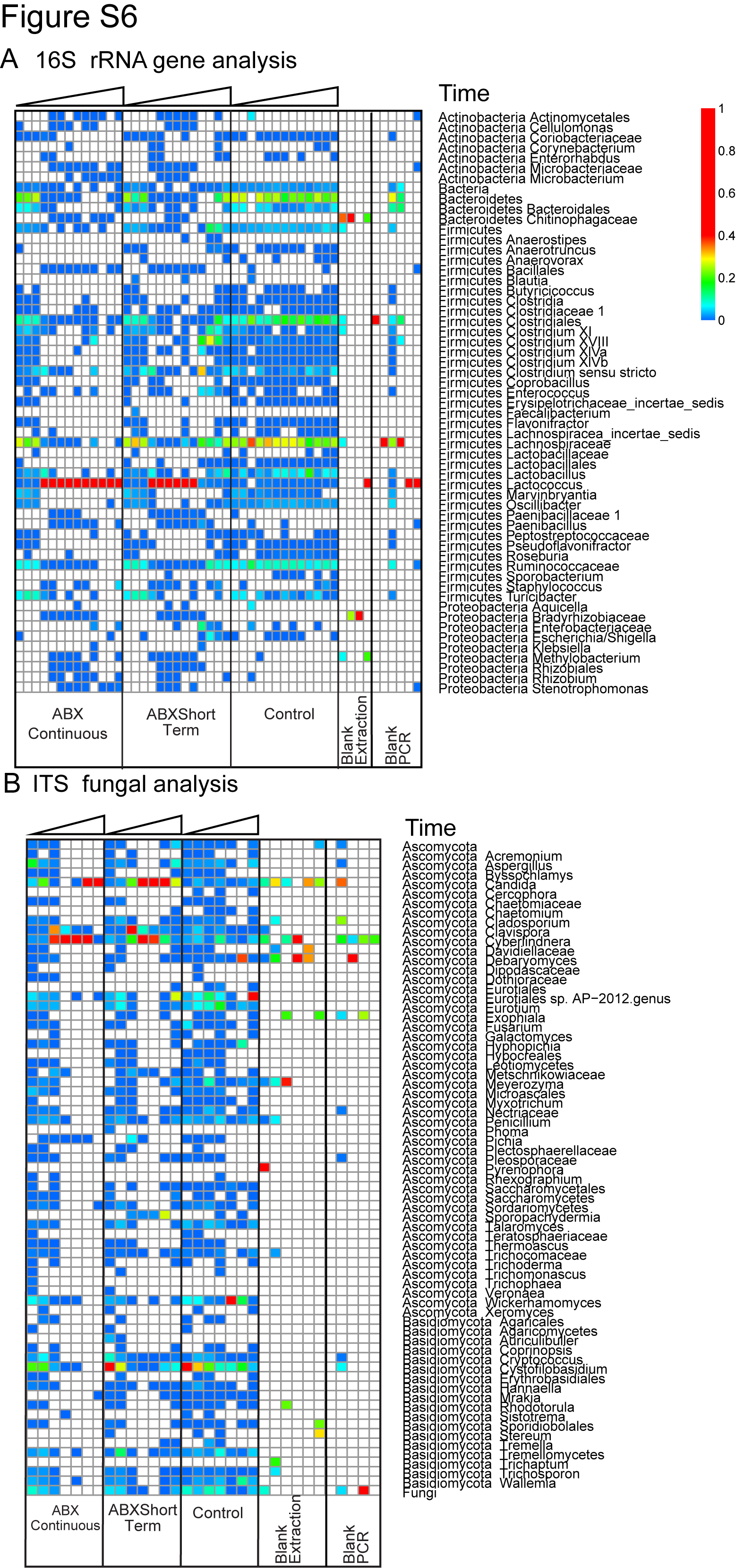

Supplement: Figure S6 — Comparison of contamination controls to experimental samples for the 16S A) and ITS B) amplicons. “Extraction control” indicates sequences derived from blank purifications using DNA-free water. Each column showing mouse data is an average over all reads at that time point. (TIF) [file pone.0071806.s006.tif]
